# Development of a penem antibiotic against *Mycobacteroides abscessus*

**DOI:** 10.1038/s42003-020-01475-2

**Published:** 2020-12-07

**Authors:** Hunter R. Batchelder, Elizabeth Story-Roller, Evan P. Lloyd, Amit Kaushik, Kristina M. Bigelow, Emily C. Maggioncalda, Eric L. Nuermberger, Gyanu Lamichhane, Craig A. Townsend

**Affiliations:** 1 Department of Chemistry, Johns Hopkins University, Baltimore, MD 21218 USA; 2 Center for Tuberculosis Research, Division of Infectious Diseases, Johns Hopkins University School of Medicine, Baltimore, MD 21287 USA

**Keywords:** Medicinal chemistry, Antimicrobials

## Abstract

β-lactams are the most widely used antibiotic class to treat bacterial infections in humans. *Mycobacteroides abscessus* is an emerging pulmonary pathogen resistant to most antibiotics, including penicillins and cephalosporins. With no current FDA-approved treatment and cure rates <50%, there is a pressing need for effective therapies. Here we report T405, a new β-lactam of the penem subclass that exhibits potent activity against *M. abscessus* and a panel of drug-resistant strains isolated from cystic fibrosis patients. Additionally, in combination with the β-lactamase inhibitor avibactam, the rate of spontaneous resistance of *M. abscessus* to T405 approached the limit of detection. Lastly, we show the favorable pharmacokinetic profile of T405 in mice and the absence of toxicity at elevated dosage, which support the clinical potential of this compound.

## Abstract (web summary)

Batchelder et al. report a new penem class antibiotic, T405, which exhibits potent activity against *M. abscessus* and clinical isolates from cystic fibrosis patients. The development of resistance to T405 is inhibited with the addition of a β-lactamase inhibitor, avibactam. Its clinical potential is further demonstrated by T405 displaying a favourable pharmacokinetic profile in mice with an absence of toxicity.

## Introduction

In patients with structural lung diseases, such as bronchiectasis or cystic fibrosis, infections with non-tuberculous mycobacteria are often viewed as the most difficult invasive pulmonary infections to treat^1,2^. Of these organisms, the rapidly growing *Mycobacteroides abscessus* is considered to be the most virulent, with inherent and growing spontaneous resistance to available antibiotics^3^. Current therapeutic guidelines for *M. abscessus* infections require at least 12–18 months of multidrug therapy that includes 2–4 months of treatment induction with a macrolide antibiotic and at least two parenteral drugs, often amikacin with a β-lactam—imipenem or cefoxitin^4,5^. Despite this prolonged treatment, sputum culture conversion rates as low as 25% and cure rates of 30–50% in patients with pulmonary *M. abscessus* infection^6,7^ underscore the need for new antibiotics with improved activity. For these reasons, *M. abscessus* has been labeled “a new antibiotic nightmare” and “environmental bacterium turned clinical nightmare”^3,8^.

**Fig. 1:**
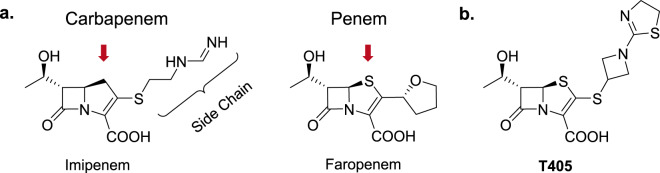
Carbapenem and penem β-lactam antibiotics. **a** Structures of the carbapenem Imipenem and the penem Faropenem are shown with red arrows indicating the differences in the ring structures. **b** Structure of the new penem developed here, T405.

β-Lactam antibiotics exert their antimicrobial effect by inhibiting two classes of enzymes that catalyze the final step of peptidoglycan synthesis in the bacterial cell wall: d,d-transpeptidases (also known as penicillin-binding proteins) and l,d-transpeptidases. In *M. abscessus*, linkages generated by l,d-transpeptidases predominate in the cell wall peptidoglycan^9^ and are inhibited strongly by the carbapenem and penem subclasses of β-lactams^10–12^. Much like carbapenems, penems contain a substrate-like bicyclic core in which the unsaturated, five-member ring causes strain on the β-lactam ring, which enhances its reactivity. The slightly longer C–S bonds of the penem nucleus (red arrows, Fig. 1), however, both reduce their intrinsic ring strain and confer greater hydrolytic stability. Carbapenems are often considered as the last line of defense against many difficult-to-treat bacterial infections. This critical property has resulted in many iterations of carbapenems to become commercially available with a focus on sidechain modification (indicated in Fig. 1) to improve inactivation of d,d-transpeptidases and resistance to hydrolysis by continually evolving microbial β-lactamases. This commercial effort to develop carbapenems contrasts with the penem subclass, of which faropenem is presently the only marketed penem, with availability in a few countries. The recent, surprising finding that among all β-lactam subclasses, faropenem is the most potent and selective inhibitor of l,d-transpeptidases of several bacteria, including *M. tuberculosis* and *M. abscessus*, provided inspiration to reassess the potential of the penem subclass to develop new therapeutics against these bacteria^11^. Herein, we describe the synthesis and development of a new penem, T405, and its potent activity against clinical isolates of *M. abscessus*. T405 shows no detectable toxicity in mice at doses up to 300 mg/kg, and only low induction of resistance.

## Results

### Identification and antimicrobial analysis of new penem antibiotic

Initial antimicrobial activity of T405 was identified from a screen of a library of penems synthesized in-house that built upon the penem core while varying the sidechains branching from C2 of the carbon–carbon double bond. The minimum inhibitory concentration (MIC) of T405 against *Mycobacterium tuberculosis* reference strain H_37_Rv of 0.5–1 µg/mL is similar to that of meropenem^13^, the carbapenem currently recommended for treating select drug-resistant tuberculosis. Next, we determined the MIC of T405 against *M. abscessus* reference strain ATCC 19977, and found it to be 2 μg/mL. The MIC_90_ of imipenem and cefoxitin, the β-lactams currently recommended as the standard treatment for *M. abscessus* infections^5^, are 16–32 and 32–64 μg/mL, respectively^14^. The in vitro potency of T405, therefore, greatly exceeds that of the β-lactams currently used to treat *M. abscessus* infection.

*M. abscessus* strains isolated from patients in the clinic display a wide range of antibiotic susceptibilities. Therefore, MICs of T405 were determined for 20 independent clinical *M. abscessus* strains isolated from cystic fibrosis patients at the Johns Hopkins Hospital^15,16^. MICs against these strains ranged from 1 to 8 µg/mL (Table 1), with 17/20 strains exhibiting MICs of ≤4 µg/mL (MIC_50_ 2 µg/mL), which compare favorably to the *M. abscessus* MIC breakpoints for imipenem of ≤4 µg/mL (susceptible) and 8 µg/mL (intermediate)^17^. Compared to the imipenem MIC_50_ and MIC_90_ of 16 and 32 µg/mL, respectively, and the faropenem MIC_50_ and MIC_90_ of 512 and 512 µg/mL, respectively, against these strains, the T405 MIC_50_ and MIC_90_ of 2 and 8 µg/mL, respectively (Table 1), indicate superior potency of T405 against *M. abscessus*.

**Table 1:** MICs of T405, imipenem and faropenem tested against the reference strain (ATCC 19977) and 20 clinical strains of *M. abscessus* in vitro.

Isolate #	T405	Imipenem	Faropenem
MIC (μg/ml)			
ATCC 19977	2	8	512
1N	2	8	256
2N	2	8	256
3N	2	8	256
4N	1	16	256
5N	1	16	512
11N	8	32	512
13N	1	8	64
14N	8	32	512
202	2	16	512
203	2	32	128
204	8	32	512
214	1	8	128
215	2	16	512
JHH2	4	32	512
JHH4	2	16	512
JHH9	4	16	512
8N	4	32	512
JHHKB	2	256	512
JH1801	1	16	512
JH1802	4	16	256

MIC_50_ and MIC_90_ of imipenem are 16 and 32 µg/mL and for faropenem are 512 and 512 µg/mL, respectively.

Another consideration for developing a new β-lactam antibiotic against *M. abscessus* is the activity of natively encoded β-lactamase Bla_Mab_. This enzyme hydrolyzes many β-lactam antibiotics currently used to treat bacterial infections and, therefore, contributes to its inherent resistance to many antibiotics in the class^18^. Co-administration of the β-lactamase inhibitor avibactam inhibits Bla_Mab_ and can reduce the MIC of β-lactam antibiotics with initially high MICs^15,19–21^. To assess if avibactam potentiates the activity of T405 against *M. abscessus*, we determined the MIC of T405 in combination with avibactam against reference strain ATCC 19977 and 10 clinical isolates. A fixed concentration of avibactam, 4 µg/mL, was used as this clinically relevant concentration has been reported in prior studies^15,20^. Addition of 4 µg/mL avibactam did not reduce the T405 MIC against 2 strains and reduced the T405 MIC against the remaining nine strains by only two-fold (7 strains) or fourfold (2 strains) (SI Table 1). In contrast to many commercially available β-lactams that have greater reductions of their MIC in the presence of a β-lactamase inhibitor, the potency of T405 does not appear to be greatly affected by Bla_Mab_.

The development of resistance is a concern for any new potential drug. Antibiotics or drug combinations that minimize its emergence may have greater longevity in the clinic. We determined the frequency of selection of spontaneous resistant mutants of *M. abscessus* in the presence of T405. The frequency of T405 resistance when tested at the MIC (2 µg/mL) against ATCC 19977 was similar to that of the first-line drug isoniazid against *M*. *tuberculosis*^22^, and approximately 1 log higher than that for imipenem at its MIC (8 µg/mL) (Table 2). However, when combined with avibactam at its MIC of 256 µg/mL, a nearly 3-log reduction in the frequency of resistance was observed (5.8 × 10^−9^).

**Table 2:** Frequency of spontaneous resistant mutants of *M. abscessus* ATCC 19977 recovered against T405 and imipenem at their individual MICs and when the T405 MIC is combined with avibactam at its individual MIC.

	Imipenem	T405	T405 + avibactam
Resistance frequency	1.9 × 10^−7^	2.0 × 10^−6^	5.8 × 10^−9^

### Pharmacokinetics profile of T405

**Fig. 2:**
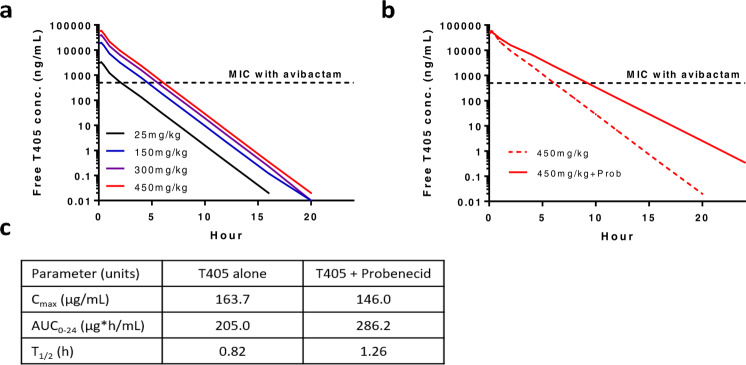
Simulated single-dose plasma-free T405 concentration–time profiles in BALB/c mice. **a** Escalating doses of T405 administered alone. **b** 450 mg/kg dose of T405 administered alone and in combination with probenecid. **c** Single-dose plasma PK parameters for total T405 concentration in mice after subcutaneous injection of 25 mg/kg (*n* = 3 mice per time point per arm).

Based on the exceptional antimicrobial activity displayed by T405 against a panel of variably drug-resistant clinical isolates of *M. abscessus* and the knowledge that T405 was well tolerated when administered subcutaneously to mice at a dose of 300 mg/kg twice daily for 21 days in two prior mouse efficacy studies against another pathogen (*n* = 5), the pharmacokinetic (PK) profile was investigated in mice. Mice were dosed subcutaneously with T405 at 25 mg/kg, with and without the renal excretion inhibitor probenecid or the human dehydropeptidase inhibitor cilastatin, and plasma samples were analyzed for T405 concentration by mass spectrometry (SI Table 3). A one-compartment model best fit the data. Plasma PK parameters are shown in Fig. 1c. The half-life was 0.82 h when dosed alone and 1.26 h when dosed with probenecid, both longer than half-lives in mice of 0.19 h reported for faropenem^23^ and 0.34–0.51 h reported for the carbapenems imipenem, meropenem, biapenem, and doripenem, each dosed with cilastatin^24^. The half-life of T405 was not affected by co-administration with cilastatin. The blood plasma binding of the compound was assessed using rapid-equilibrium dialysis to be 98% bound to mouse serum^25^. PK simulations indicated the potential for in vivo efficacy against *M. abscessus* infection in mice. When a dose of 450 mg/kg twice daily in mice was simulated, assuming a plasma-free drug fraction of 2%, the predicted free drug time above MIC (fT_>MIC_) value was 48% for an MIC of 0.5 µg/mL observed against the ATCC 19977 strain in the presence of avibactam and 35% for an MIC of 2 µg/mL observed in the absence of avibactam (also the MIC_50_ against the clinical strains in Table 1) (Fig. 2a). fT_>MIC_ remained above 40% with the 300 mg/kg twice daily dose in the presence of avibactam. Upon co-administration of 450 mg/kg twice daily with probenecid, the predicted fT_>MIC_ increased to 72 and 54% in the presence and absence of avibactam, respectively (Fig. 2b). For other bacterial pathogens, carbapenem free-drug concentrations exceeding the MIC for 20 and 40% of the dosing interval are associated with bacteriostatic and bactericidal effects, respectively^16^.

## Discussion

T405 has excellent antimicrobial activity against *M. abscessus*, yielding the lowest MICs reported for a β-lactam against this pathogen to date. The MIC_50_ and MIC_90_ of T405 are significantly lower compared to that of imipenem and cefoxitin, two β-lactams that are currently recommended as first-line treatment for *M. abscessus* infections^5^. Moreover, the compound’s activity against diverse clinical isolates indicates that it may be effective against resistant *M. abscessus* strains. When tested in combination with the β-lactamase inhibitor avibactam, the MIC was not greatly improved as has been observed with other β-lactams, which suggests that T405 is likely a poor substrate of β-lactamase(s) present in *M. abscessus*. We determined the spontaneous frequency of *M. abscessus* ATCC 19977 CFU resistant to T405 at 2 μg/mL (its MIC when used alone) with and without avibactam. Intriguingly, the addition of avibactam to T405 resulted in a 3-log reduction in the observed frequency of resistance. This outcome is likely related to avibactam increasing the effective concentration of T405, as is apparent from the MIC data (SI Table 1), and thereby preventing the growth of a subpopulation of bacilli that are spontaneously resistant to T405, but only at concentrations equal to its MIC (when tested alone) or a small multiple of that MIC. If this finding is supported by further studies testing T405 with lower, more clinically relevant concentrations of avibactam or another β-lactamase inhibitor, it may indicate that such a combination could reduce the risk of selection of T405-resistant mutants. T405 also displayed a favorable half-life in mice that is most likely due to high protein binding, suggesting that it may be a good candidate for less frequent dosing. Our study demonstrates that there is yet untapped potential for the further development of β-lactams. The new penem reported here exemplifies such an agent with promise for further pre-clinical development to treat drug-resistant *M. abscessus* disease.

## Methods

### Bacterial strains and in vitro growth conditions

The *M. abscessus* reference strain ATCC 19977 (ATCC, Manassas, VA) as well as 20 deidentified clinical strains obtained from patients at the Johns Hopkins Hospital^15,16^ were used for this study. The clinical isolates have been identified to the sub-species level^26^ and the MICs of many other β-lactams against these strains have been published^15,21^. The strains were grown in Middlebrook 7H9 broth (Difco) supplemented with 0.5% glycerol, 10% albumin-dextrose-NaCl enrichment, and 0.05% Tween-80, at 37 °C with constant shaking at 220 RPM in an orbital shaker. Imipenem, faropenem, and avibactam were obtained from Sigma-Aldrich.

### Minimum inhibitory concentration

MIC of each drug against *M. abscessus* was determined using the broth dilution method^27^ in accordance with CLSI guidelines specific for this organism^17^, except that Middlebrook 7H9 broth was used instead of cation-adjusted Mueller–Hinton broth^16^. Powdered drug stocks were reconstituted in dimethyl sulfoxide and twofold serial dilutions were prepared in Middlebrook 7H9 broth to obtain final drug concentrations ranging from 512 to 0.5 µg/mL in 96-well plates. 10^5^ colony forming units (CFU)/mL of *M. abscessus* from an exponentially growing culture were added to each well. *M. abscessus* cultured without drug and 7H9 broth alone were included in each plate as positive and negative controls, respectively. Plates were incubated at 30 °C for 72 h in accordance to CLSI guidelines for MIC determination for *M. abscessus*^17^. Growth of *M. abscessus* or lack thereof was assessed by visual inspection and an MIC for each drug was recorded as the lowest concentration at which *M. abscessus* growth was not observed. Results are reported as medians determined from three independent biological replicates. Similarly, to determine the MIC of T405 against *M. tuberculosis*, strain H_37_Rv was used and was based on the broth dilution method described above according to CLSI guidelines. Middlebrook 7H9 broth was used for this assay and growth or lack thereof of *M. tuberculosis* was assessed after incubation at 37 °C for 14 days.

### Determination of frequency of selection for spontaneous drug resistance

The CFU/mL of *M. abscessus* in culture at an *A*_600nm_ of 1.0 was initially determined as follows. *M. abscessus* was grown to exponential phase, adjusted to an *A*_600nm_ of 1.0 in Middlebrook 7H9 broth and was serially diluted 10-fold in this broth. One hundred microliters of each dilution was plated onto Middlebrook 7H10 agar, which were incubated at 37 °C for 72 h. Resultant CFU counts were used to determine *M. abscessus* CFU density in culture. This assessment was repeated three times and the mean *M. abscessus* CFU density was used in calculations in subsequent experiments.

To determine frequency of spontaneous drug resistance emergence, 10 mL of *M. abscessus* culture grown to exponential phase in 7H9 broth was used to prepare a suspension at *A*_600nm_ of 1.0, and 1.0 mL of this suspension was inoculated onto each of 10 total Middlebrook 7H10 agar plates, which were supplemented with either T405 or a combination of T405 and avibactam. This experiment was concurrently performed using imipenem for comparison. These assessments were performed at the respective MICs of each drug to promote selection of resistant mutants. CFU were counted after 7 days of incubation at 37 °C. The frequency of drug-resistant mutants was determined from the number of spontaneous mutants observed as a percentage of the input CFU inoculum.

### Mouse plasma protein binding assay

The plasma protein binding assay was performed with a Rapid Equilibrium Dialysis (RED) device (Thermo Scientific, Rockford, IL) according to the manufacturer’s directions and as previously described^25^. T405 stability in mouse plasma was first tested in triplicate to ensure it would not affect plasma protein binding measurement. T405 (5 μM) was incubated with mouse plasma at 37 °C. Aliquots were taken at 30 min and 4 h, diluted with phosphate-buffered saline (PBS), and quenched in 75 µL cold acetonitrile/water (9:1) + 0.1% formic acid mixture containing 5 µM internal standard 1,8-diazabicyclo(5.4.0)undec-7-ene (DBU). The resulting mixture was incubated at −80 °C for 30 min and subjected to centrifugation at 20,000 × *g* for 20 min. The supernatant was analyzed by UPLC-MS for changes in T405 concentration. Upon discovery of no measurable degradation of T405 in mouse plasma over 4 h, the mouse plasma protein binding of T405 was tested using the RED device in triplicate. Using PBS in the buffer well and mouse plasma in the sample well, the relative T405 concentrations were determined after 4 h as previously described in the plasma stability assay.

### Mouse pharmacokinetics

All procedures involving mice were approved by the Animal Care and Use Committee of Johns Hopkins University. T405 was dissolved in PBS. Uninfected female BALB/c mice, aged 6–7 weeks, were administered a single 25 mg/kg dose of T405 in 100 µL subcutaneously, either alone or in combination with probenecid (250 mg/kg) or cilastatin (25 mg/kg). Blood was sampled from three mice per group per time point by mandibular vein puncture or cardiac puncture at the following time points: 5, 15, 30, 60, 120, and 240 min post-dose. Plasma was separated in heparin-containing tubes and frozen. T405 concentrations in plasma were determined using a validated LC/MS-MS method developed in the Analytical Pharmacology Core of the Sidney Kimmel Comprehensive Cancer Center at Johns Hopkins University School of Medicine. Concentration–time data were analyzed by standard non-compartmental and compartmental techniques using WinNonlin (version 7.0; Pharsight, Mountain View, CA). A one-compartmental model was used to perform simulations to predict the plasma concentration–time profile after subcutaneous doses of 150, 300, and 450 mg/kg, alone and in combination with probenecid. A plasma free drug fraction of 2% of the total drug concentration was included in the simulations.

## Supplementary information

Supplementary Information

## Data Availability

The plasma drug concentration datasets generated and analyzed during the current study are available from the corresponding author on reasonable request.
