# Supplementary material for: Development of a penem antibiotic against Mycobacteroides abscessus
Source: Commun Biol. 2020 Dec 7;3:741. doi: 10.1038/s42003-020-01475-2 (PMC7721803; doi:10.1038/s42003-020-01475-2)

Supplementary Information

General Methods and Instrumentation

All reagents and starting materials were purchased and used without further purification unless otherwise indicated. Anhydrous solvents were dried using an LC Technology Solutions (Salisbury, MA) SPBT-1 solvent purification system. Silica gel chromatography was performed using Sorbtech Silica Gel (60 Å, 40-75mm particle size) or RediSep Rf disposable flash columns (60 Å, 40-63 µm irregular particle size) on a Teledyne ISCO (Lincoln, NE) CombiFlash EZ Prep. Preparative HPLC was carried out on the same instrument outfitted with a Phenomenex (Torrance, CA) Luna 10µ C18(2) 100 Å column (250 × 21.20 mm ID). All ^{1}H- and ^{13}C-NMR spectra were recorded on a Bruker (Billerica, MA) UltraShield 400 MHz or 300 MHz Avance spectrometer. The Johns Hopkins Chemistry Department Mass Spectrometry Facility determined exact masses by fast-atom bombardment (FAB) on a VG 70S/E instrument (Danvers, MA) or by high resolution ultra-performance liquid chromatography–electrospray ionization mass spectrometry (UPLC-ESIMS) using a Waters (Milford, MA) Acquity/Xevo-G2.

MIC with avibactam

	MIC (ug/ml)	
Isolate #	T405	T405 + Avibactam
ATCC 19977	2	0.5
5N	1	0.5
14N	8	4
202	2	0.5
204	8	8
214	1	0.5
215	2	1
JHH4	2	1
JHHKB	2	1
JH1801	1	0.5
JH1802	4	4

SI Table 1 | MICs of **T405** with and without the addition of avibactam (4 µg/mL) against the *M. abscessus* reference strain (ATCC 19977) and a subset of clinical strains *in vitro.*

	T405 concentration (ng/ml)								
Hour	T405			T405+Prob			T405+Cila		
0.083333	169	139	160	128	132	144	116	98.7	135
0.25	157	172	162	106	186	146	171	103	84.7
0.5	143	133	90.9	138	116	118	100	101	99.1
1	65	65.6	51.3	84.8	96.8	65.6	64.6	56.3	73.2
2	25.1	24.6	29.2	37.6	57.4	38.5	17.6	24.6	22.9
4	4.56	4.39	10.8	17.7	22.9	14.7	4.2	6.2	7.08

SI Table 2 | T405 concentration in blood over 4 h when administered in a single 25 mg/kg dose alone, with probenecid, or with cilastatin.

	T405 concentration (ng/ml) by dose			
Hour	25mg/kg	150mg/kg	300mg/kg	450mg/kg
0.083	3120	18720	37440	56160
0.25	3273.33	19640	39280	58920
0.5	2446	14676	29352	44028
1	1212.67	7276	14552	21828
2	526	3156	6312	9468
4	131.67	790	1580	2370
6	29.76	178.58	357.16	535.74
8	6.87	41.2	82.4	123.6
10	1.58	9.51	19.01	28.52
12	0.37	2.19	4.39	6.58
16	0.02	0.12	0.23	0.35
20	0	0.01	0.01	0.02
24	0	0	0	0

SI Table 3 | PK simulation of escalating doses from 25 mg/kg to 450 mg/kg of **T405** alone. Data are represented in Figure 2a.

	T405 concentration (ng/ml)	
Hour	450mg/kg	450mg/kg+Prob
0.083	56160	48480
0.25	58920	52560
0.5	44028	44640
1	21828	29664
2	9468	16020
4	2370	6636
6	535.74	2425.1
8	123.6	908.73
10	28.52	340.52
12	6.58	127.6
16	0.35	17.92
20	0.02	2.52
24	0	0.35

SI table 4 | PK simulation of 450 mg/kg dose of **T405** alone and with probenecid. Data are represented in Figure 2b.

Synthesis of T405

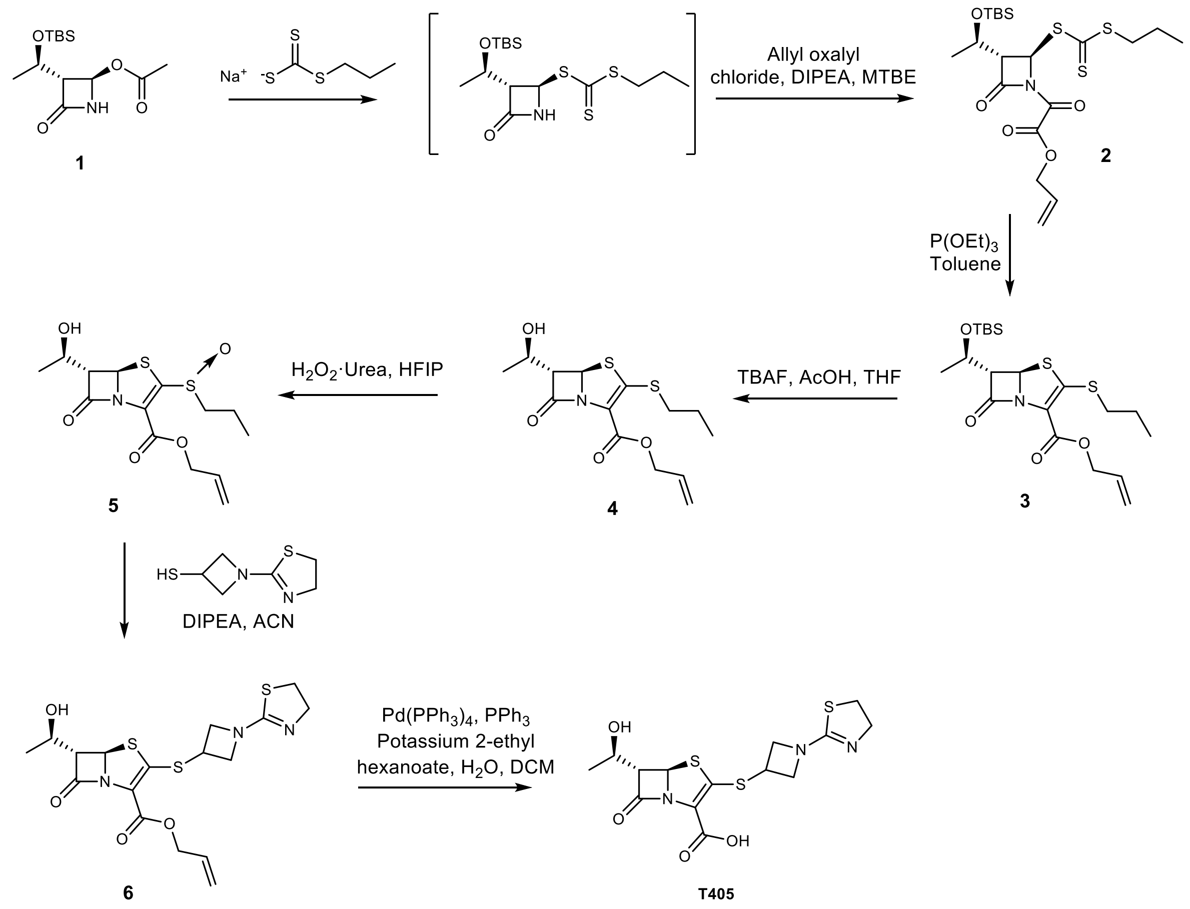

Allyl 2-((3*R*,4*S*)-3-((*R*)-1-((*tert*-butyldimethylsilyl)oxy)ethyl)-2-oxo-4-(propylthio-carbonothioyl-thio)azetidin-1-yl)-2-oxoacetate (2). Procedure used was adapted from a previous report[1]. To a suspension of 60% sodium hydride in mineral oil (14 g, 350 mmol) in methyl *tert*-butyl ether (MTBE) (500 mL), a solution of 1-propanethiol (20.7 mL, 223 mmol) in MTBE (100 mL) was slowly added under nitrogen and then stirred for 1 h. Carbon disulfide (20.93 mL, 348 mmol) was then added to the mixture and left to react for an additional 1 h. Magnesium sulfate (3 g) was then added to the mixture and the slurry was vacuum filtered through a pad of Celite under nitrogen (WARNING: the retentate is flammable when in contact with moisture), and the retentate was washed twice with 100 mL of MTBE. To the filtrate (3*R,*4*R*)-4-acetoxy-3-[(*R*)-1-(*tert*-butyldimethylsilyloxy)ethyl]azetidin-2-one (**1**) (50 g, 174 mmol) was added portion-wise. The slurry was allowed to react for 1 h at which point it was filtered

through a pad of Celite and the retentate was washed twice with 100 mL of MTBE. The filtrate was then cooled to -10 °C to which allyl oxalyl chloride (25 mL, 204 mmol) was added dropwise and immediately followed by the dropwise addition of TEA (28 mL, 200 mmol). The reaction mixture was allowed to warm to room temperature and stirred for 1 h before it was quenched with water (100 mL). The organic layer was washed with sodium bicarbonate (100 mL), dried with anhydrous sodium sulfate, filtered, and concentrated. The product **2** was crystallized from MTBE and hexanes to provide yellow needles (45.8 g, 53%). **2:** ^{1}H NMR (400MHz ,$CDCl_3$) δ = 6.76 (dd, *J* = 0.4, 3.5 Hz, 1H), 5.94 (tdd, *J* = 6.1, 10.6, 17.0 Hz, 1H), 5.39 (qd, *J* = 1.4, 17.0 Hz, 1H), 5.31 (qd, *J* = 1.0, 10.4 Hz, 1H), 4.78 (qd, *J* = 1.2, 6.1 Hz, 2H), 4.39 (dq, *J* = 2.5, 6.3 Hz, 1H), 3.56 (dd, *J* = 2.5, 3.5 Hz, 1H), 3.39 (dt, *J* = 3.7, 7.2 Hz, 2H), 1.76 (sxt, *J* = 7.4 Hz, 3H), 1.24 (d, *J* = 6.5 Hz, 3H), 1.03 (t, *J* = 7.4 Hz, 3H), 0.86 (s, 9H), 0.09 (s, 3H), 0.06 (s, 3H). ^{13}C NMR (101MHz, $CDCl_3$) δ = 218.3, 163.5, 159.1, 154.4, 130.4, 120.2, 67.4, 66.0, 64.5, 58.9, 38.9, 26.9, 25.6, 21.9, 21.3, 17.8, 13.4, -4.3, -5.3. HRMS (FAB), $C_{20}H_{34}NO_5S_3Si$ [$M+H^+$] calculated: 492.1368; found: 492.1370. ^{1}H and ^{13}C NMR are identical to the characterization data reported in the literature[1].

Allyl (5*S*,6*R*)-6-((*R*)-1-((*tert*-butyldimethylsilyl)oxy)ethyl)-7-oxo-3-(propylthio)-4-thia-1-azabicyclo[3.2.0]hept-2-ene-2-carboxylate (3). The method of preparation was adapted from the literature.[1] An oven-dried flask was charged with **2** (10 g, 20.3 mmol) and toluene (300 mL), fitted with a reflux condenser, and stirred under nitrogen. The mixture was brought up to 90 °C and stirred vigorously at which point triethylphosphite (30% w/w in toluene, 93.5 mmol) was added dropwise over 2 h. The reaction was allowed to proceed for another 2 h before the reaction mixture was concentrated at 50 °C *in vacuo*. The solution was washed with aqueous 0.1N HCl (50 mL), saturated aqueous sodium bicarbonate (50 mL), and saturated sodium chloride solution (50 mL). The organic layer was concentrated *in vacuo* followed by two additions of isopropyl alcohol (50 mL) and re-concentration of the mixture. The product was crystallized from isopropyl alcohol to yield **3** (5.1 g, 57%). **3:** ^{1}H NMR (400MHz, $CDCl_3$) δ = 5.93 (tdd, *J* = 5.4, 10.7, 17.2 Hz, 1H), 5.59 (d, *J* = 1.4 Hz, 1H), 5.40 (qd, *J* = 1.6, 17.2 Hz, 1H), 5.22 (qd, *J* = 1.4, 10.4 Hz, 1H), 4.70 (tddd, *J* = 1.6, 5.5, 13.5, 28.2 Hz, 2H), 4.23 (td, *J* = 6.1, 11.5 Hz, 1H), 3.66 (dd, *J* = 1.6, 4.9 Hz, 1H), 2.91 (tdd, *J* = 7.4, 12.5, J_3 = 32.3 Hz, 2H), 1.74 (sxt, *J* = 7.4 Hz, 2H), 1.25

(d, J = 6.3 Hz, 3H), 1.03 (t, J = 7.3 Hz, 3H), 0.88 (s, 9H), 0.07 (s, 3H), 0.07 (s, 3H). ^{13}C NMR (101MHz, $CDCl_3$) δ = 172.1, 159.8, 131.9, 118.1, 117.0, 71.4, 65.3, 63.7, 37.9, 25.7, 23.3, 22.5, 17.9, 13.2, -4.3, -5.1. HRMS (FAB), $C_{20}H_{33}NO_4S_2Si$ [M^+] calculated: 443.1620; found: 443.1624. The ^{1}H and ^{13}C NMR data are identical to those reported [1].

Allyl (5*S*,6*R*)-6-((*R*)-1-hydroxyethyl)-7-oxo-3-(propylthio)-4-thia-1-azabicyclo-[3.2.0]-hept-2-ene-2-carboxylate (4). To a solution of **3** (10.2 g, 22.99 mmol) in tetrahydrofuran (THF) (64 mL) acetic acid (12.5 mL, 218 mmol) followed by *tetra-N*-butylammonium fluoride 1M solution in THF (34.5 mmol) were added under nitrogen at room temperature. The reaction mixture was stirred for 24 h and washed with saturated aqueous sodium bicarbonate. The aqueous layer was then extracted with dichloromethane (DCM) and the combined organic layers were washed with brine, dried with anhydrous sodium sulfate, filtered, and concentrated *in vacuo*. The crude product was purified by silica gel chromatography (hexanes:ethyl acetate, 1:1) and the resulting oil crystallized from ethyl acetate/hexanes to provide **4** as white needles (5.5 g, 73%). **4:** ^{1}H NMR (400MHz, $CDCl_3$) δ = 5.95 (tdd, J = 5.4, 10.6, 17.2 Hz, 1H), 5.63 (d, J = 1.6 Hz, 1H), 5.41 (qd, J = 1.5, 17.2 Hz, 1H), 5.23 (qd, J = 1.4, 10.6 Hz, 1H), 4.78 (tdd, J = 1.5, 5.4, 13.5 Hz, 1H), 4.66 (tdd, J = 1.4, 5.6, J_3 = 13.5 Hz, 1H), 4.24 (quin, J = 6.5 Hz, 1H), 3.70 (dd, J = 1.4, 6.8 Hz, 1H), 2.98 (td, J = 7.4, 12.7 Hz, 1H), 2.88 (td, J = 7.2, 12.5 Hz, 1H), 1.74 (sxt, J = 7.4 Hz, 2H), 1.35 (d, J = 6.3 Hz, 3H), 1.03 (t, J = 7.3 Hz, 3H). ^{13}C NMR (101MHz, $CDCl_3$) δ = 172.1, 159.8, 156.2, 131.9, 118.2, 116.7, 70.9, 65.5, 65.5, 64.0, 37.9, 23.3, 21.9, 13.2. HRMS (FAB), $C_{14}H_{19}NO_4S_2$ [M^+] calculated: 329.0756; found: 329.0753.

Allyl (5*S*,6*R*)-6-((*R*)-1-hydroxyethyl)-7-oxo-3-(propylsulfinyl)-4-thia-1-azabicyclo[3.2.0]hept-2-ene-2-carboxylate (5). A solution of urea•hydrogen peroxide (1.62 g, 16.7 mmol) in 1,1,1,3,3,3-hexafluoroisopropanol (16 mL) was added dropwise to a solution of **4** (5.0 g, 15.2 mmol) in 1,1,1,3,3,3-hexafluoroisopropanol (20 mL). The mixture was stirred for 24 h under nitrogen at 20-25°C. The mixture was then diluted with hexanes (50 mL) and concentrated *in vacuo*. Silica gel flash chromatography was performed (hexanes:ethyl acetate, 1:1) to yield **5** (4.63 g, 88%) as a yellow foam. Owing to instability of the product, the following reaction was performed

immediately. **5:** ^{1}H NMR (400MHz, $CDCl_3$) δ = 5.98 - 5.87 (m, 1H), 5.86 (d, *J* = 1.8 Hz, 0.33H), 5.71 (d, *J* = 1.8 Hz, 0.67H), 5.41 (qd, *J* = 1.6, 17.0 Hz, 0.33H), 5.43 (qd, *J* = 1.4, 17.2 Hz, 0.66H), 5.29 (qd, *J* = 1.2, 10.6 Hz, 1H), 4.77 (qdd, *J* = 1.6, 5.5, 13.3 Hz, 1H), 4.67 (qdd, *J* = 1.6, 5.7, 13.3 Hz, 1H), 4.27 - 4.14 (m, 1H), 3.92 (dd, *J* = 1.6, 6.8 Hz, 0.66H), 3.88 (dd, *J* = 2.0, 7.0 Hz, 0.33H), 3.21 - 3.04 (m, 1H), 2.99 (ddt, *J* = 7.0, 9.2, 13.3 Hz, 1H), 2.04 - 1.77 (m, 2H), 1.35 (d, *J* = 6.3 Hz, 3H), 1.11 (t, *J* = 7.4 Hz, 3H). ^{13}C NMR (101MHz, $CDCl_3$) δ = 171.5, 164.0, 158.2, 158.1, 130.8, 121.6, 121.4, 119.5, 119.5, 73.2, 73.2, 66.7, 65.6, 65.4, 65.4, 63.4, 57.5, 57.0, 21.8, 16.5, 16.1, 13.0. HRMS (UPLC), $C_{14}H_{20}NO_5S_2$ [M+H$^+$] calculated: 346.0777; found: 346.0783.

Allyl (5*S*,6*R*)-3-((1-(4,5-dihydrothiazol-2-yl)azetidin-3-yl)thio)-6-((*R*)-1-hydroxy-ethyl)-7-oxo-4-thia-1-azabicyclo[3.2.0]hept-2-ene-2-carboxylate (6). Diisopropylamine (5.05 mL, 29.0 mmol) was added to 1-(4,5-dihydro-2-thiazolyl)-3-azetidinethiol hydrochloride (3.66 g, 17.4 mmol) in acetonitrile (60 mL) and stirred until homogenous. The mixture was then added in one portion to a stirred solution of **5** (4 g, 11.59 mmol) in acetonitrile (81 mL) at 0 °C and stirred under nitrogen for 2 h. The solution was diluted with brine and the aqueous layer was back extracted three times with ethyl acetate. The combined organic layers were dried over anhydrous sodium sulfate, filtered, and concentrated *in vacuo*. The crude oil was purified by silica gel flash chromatography (isopropyl alcohol: DCM 1:9) to provide **6** as a yellow foam (3.1 g, 63%). **6:** ^{1}H NMR (400MHz, $CDCl_3$) δ = 5.94 (tdd, *J* = 5.4, 10.7, 17.2 Hz, 1H), 5.69 (d, *J* = 1.4 Hz, 1H), 5.41 (qd, *J* = 1.5, 17.1 Hz, 1H), 5.24 (qd, *J* = 1.2, 10.5 Hz, 1H), 4.77 (tdd, *J* = 1.4, 5.5, 13.4 Hz, 1H), 4.67 (tdd, *J* = 1.4, 5.6, 13.4 Hz, 1H), 4.40 (td, *J* = 8.4, 23.9 Hz, 3H), 4.19 (sxt, *J* = 6.5 Hz, 1H), 4.25 - 4.10 (m, 1H), 4.00 (t, *J* = 7.4 Hz, 2H), 4.06 - 3.90 (m, 2H), 3.72 (dd, *J* = 1.5, 6.9 Hz, 1H), 3.36 (t, *J* = 7.4 Hz, 2H), 1.33 (d, *J* = 6.5 Hz, 3H). ^{13}C NMR (101MHz, $CDCl_3$) δ = 172.2, 164.4, 159.5, 152.1, 131.6, 118.5, 117.6, 71.5, 65.8, 65.2, 65.0, 60.4, 59.3, 58.7, 36.2, 36.1, 21.9. HRMS (FAB), $C_{17}H_{22}N_3O_4S_3$ [M+H$^+$] calculated: 428.0773; found: 428.0765.

Potassium (5*S*,6*R*)-3-((1-(4,5-dihydrothiazol-2-yl)azetidin-3-yl)thio)-6-((*R*)-1-hydroxyethyl)-7-

oxo-4-thia-1-azabicyclo[3.2.0]hept-2-ene-2-carboxylate (T405).

The allyl ester **6** (5.3 g, 12.4 mmol), potassium 2-ethylhexanoate (2.45 g, 11.2 mmol), and tetrakis(triphenylphosphine)palladium (430 mg, 0.372 mmol) were added sequentially to vigorously stirring biphasic mixture of DCM (63 mL) and water (63 mL) under nitrogen. The reaction was allowed to proceed at 20 °C for 1 h. The layers were separated and the aqueous layer was washed three times with DCM. The aqueous product solution was then lyophilized to yield a yellow powder **T405** (3.9 g, 10 mmol) in 81% yield. The sample was then further purified by C18 high-performance liquid chromatography with a water and acetonitrile mobile phase to give a light-yellow amorphous powder. **T405:** ^{1}H NMR (400MHz, D_2O) δ = 5.71 (d, *J* = 1.0 Hz, 1H), 4.75 - 4.69 (m, 2H), 4.44 - 4.40 (m, 1H), 4.34 - 4.24 (m, 3H), 4.01 (t, *J* = 7.4 Hz, 2H), 3.94 (dd, *J* = 1.4, 6.0 Hz, 2H), 3.65 (t, *J* = 7.0 Hz, 2H), 1.30 (d, *J* = 6.4 Hz, 3H). ^{13}C NMR (101MHz, D_2O) δ = 175.4, 171.1, 166.0, 142.6, 124.6, 69.6, 64.7, 64.1, 60.5, 49.7, 35.2, 32.4, 20.1. HRMS (UPLC), $C_{14}H_{18}N_3O_4S_3$ [$M+H^+$] calculated: 388.0456; found: 388.0457.

References

1. Brenek, S. J. *et al.* Development of a practical and convergent process for the preparation of sulopenem. *Org. Process Res. Dev.* **16**, 1348–1359 (2012).

^{1}H spectrum of compound **2**

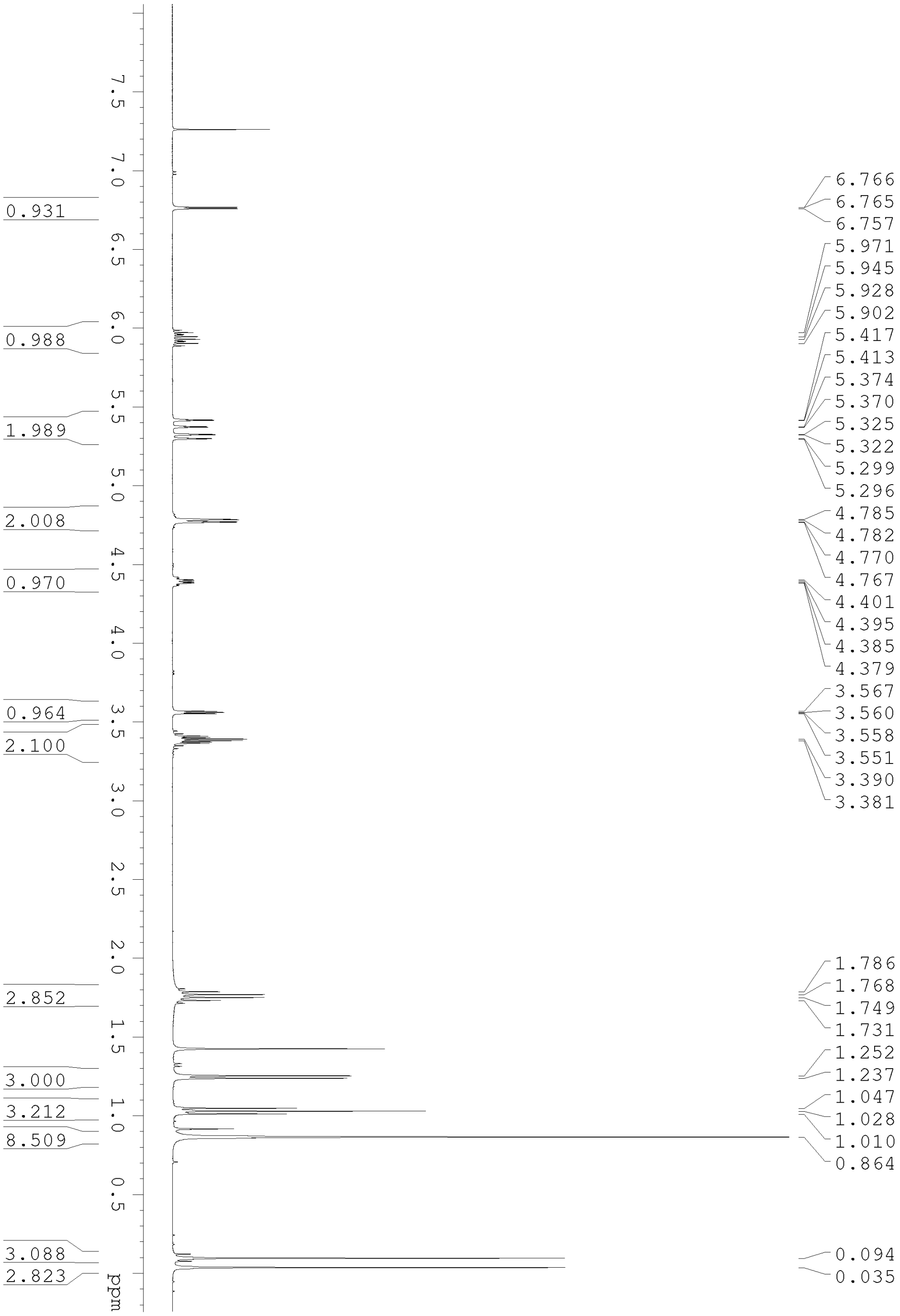

^{13}C spectrum of compound **2**

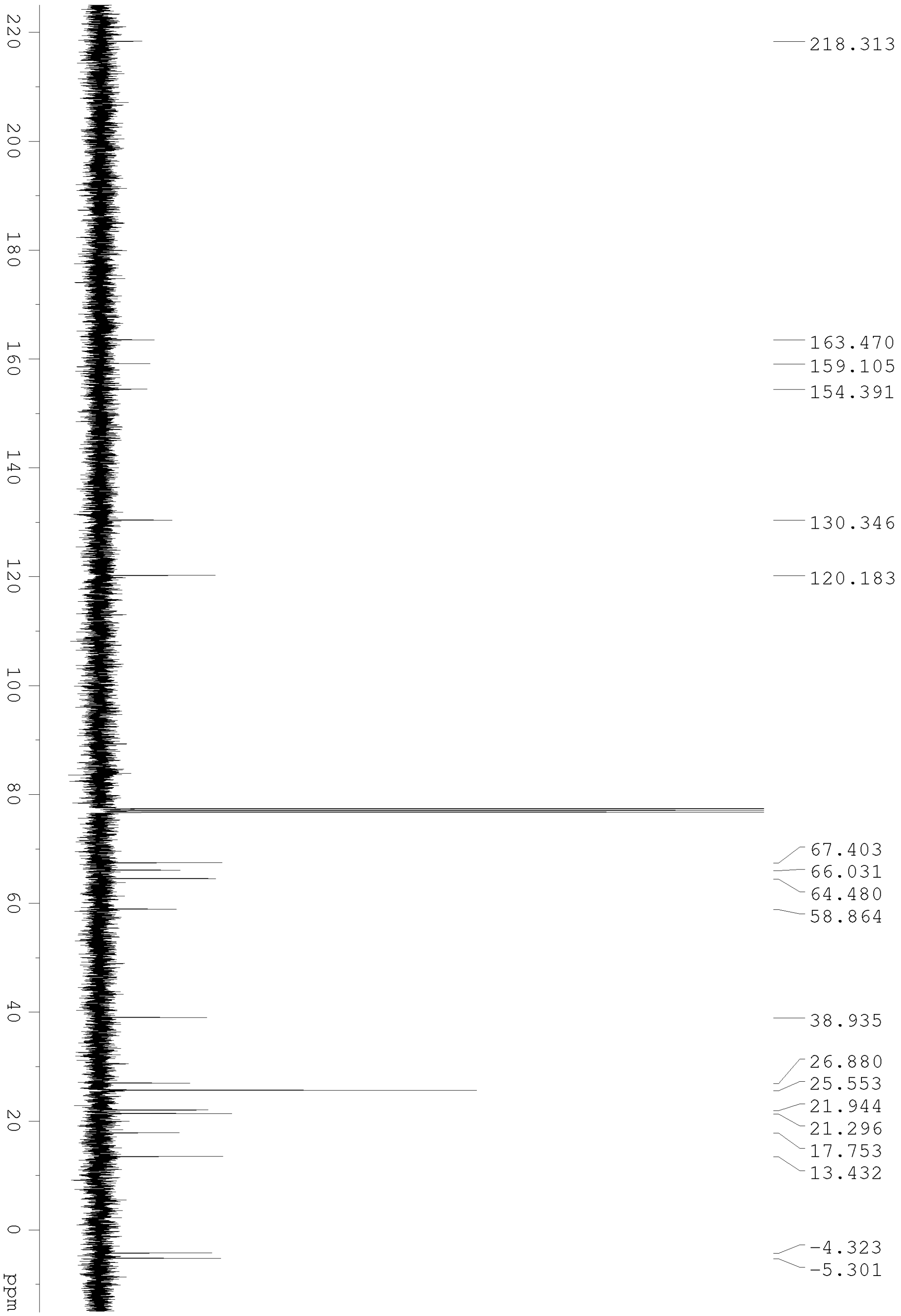

^{1}H spectrum of compound **3**

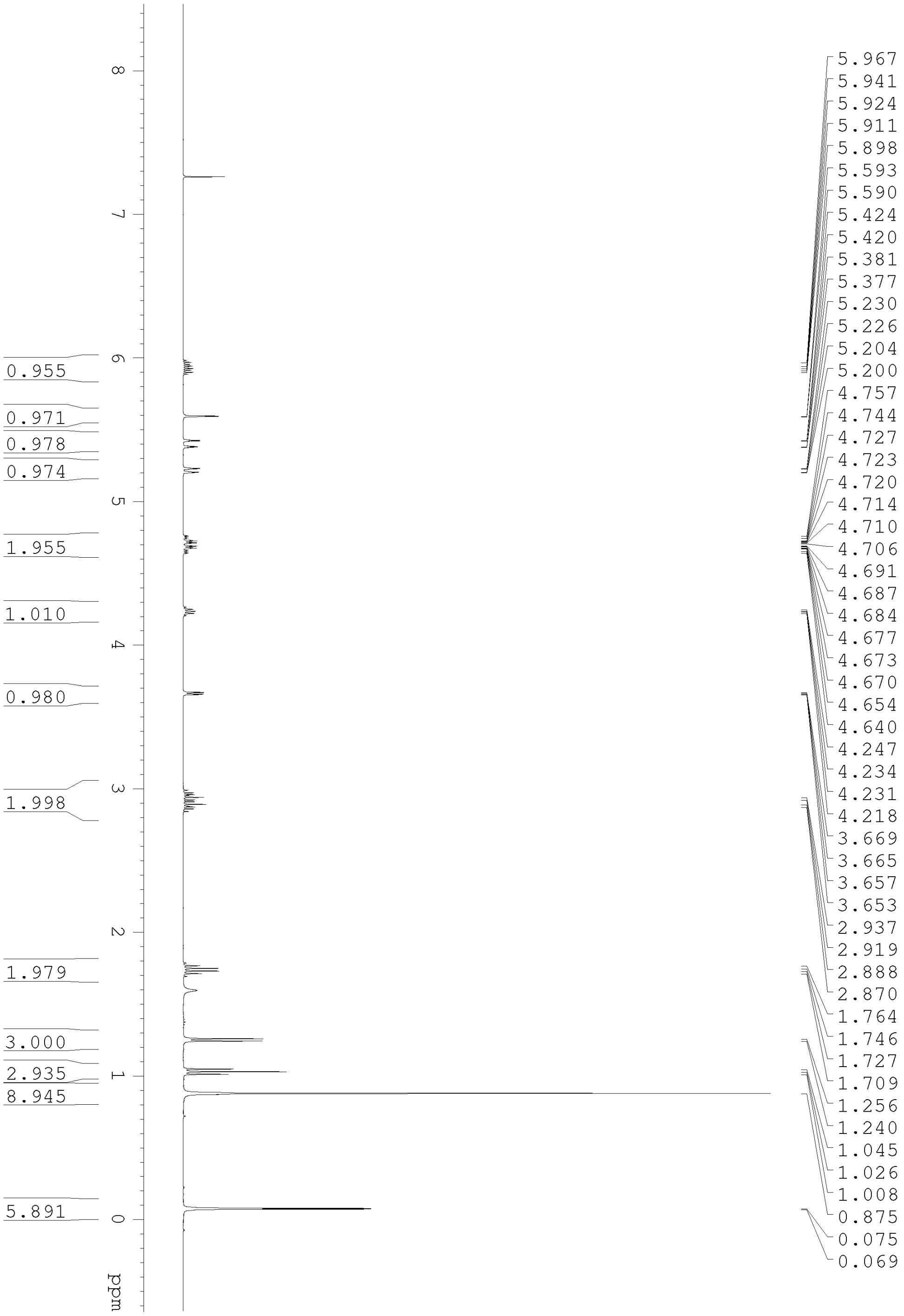

^{13}C spectrum of compound **3**

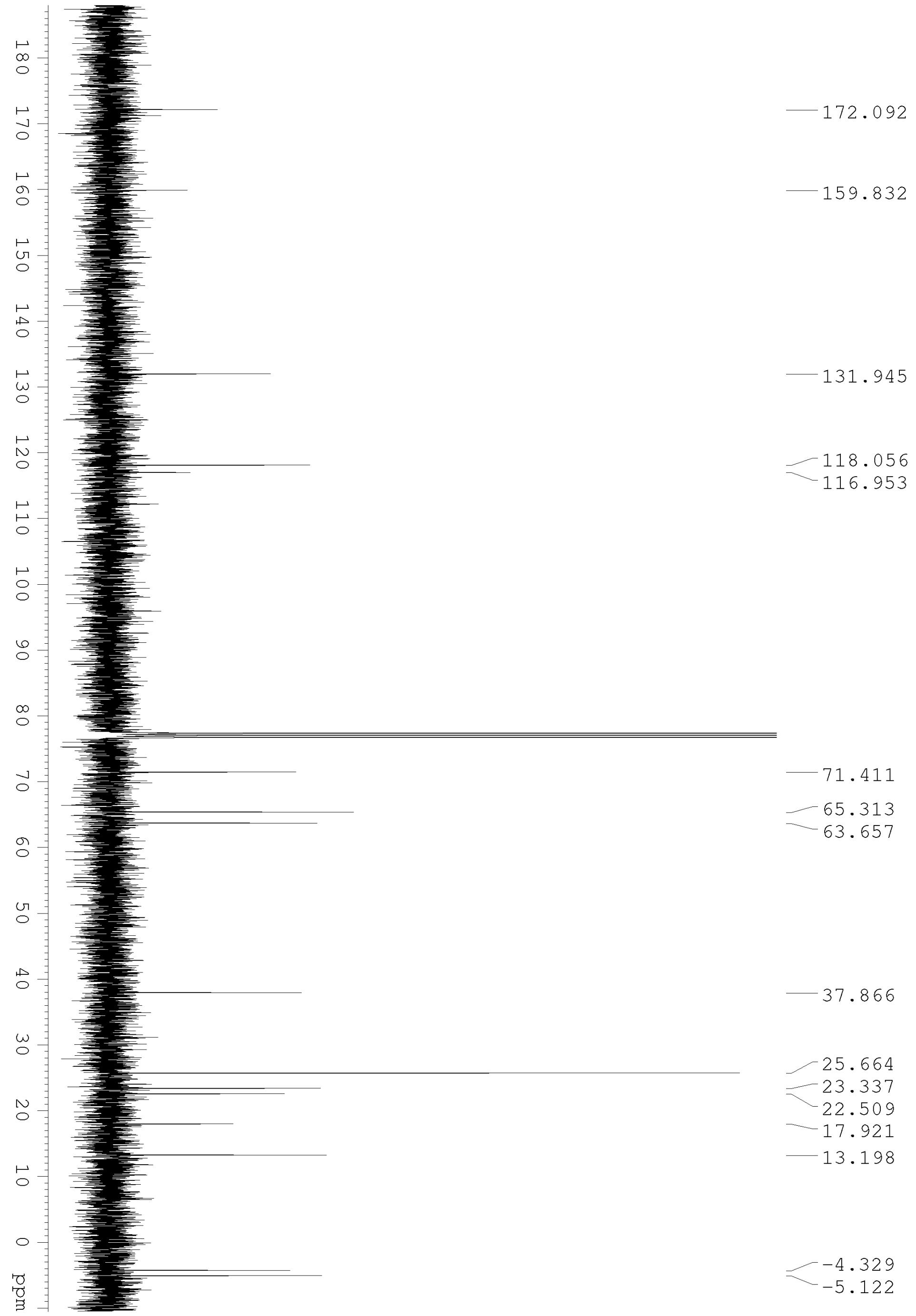

^{1}H spectrum of compound **4**

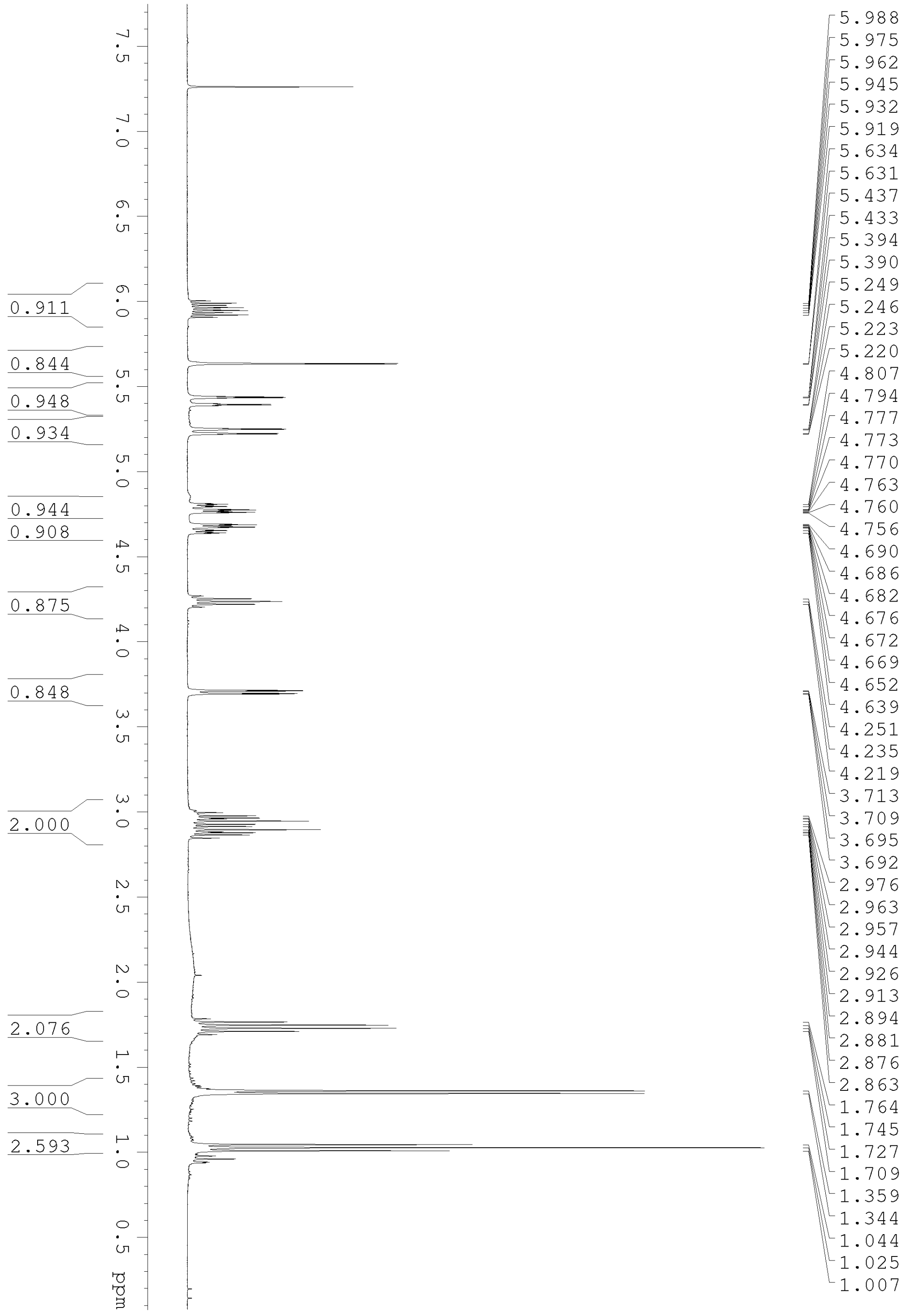

^{13}C spectrum of compound **4**

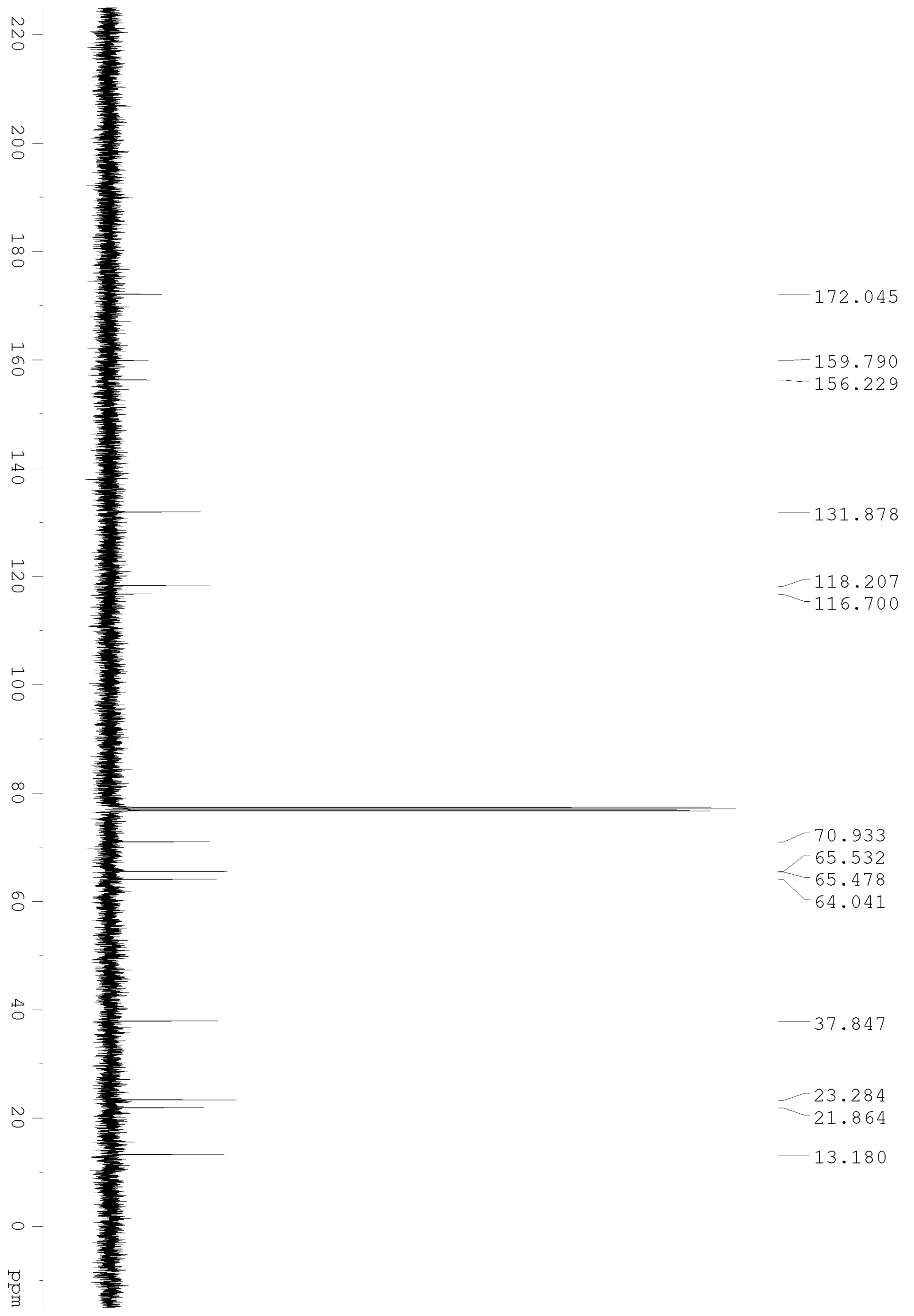

^{1}H spectrum of compound **5**

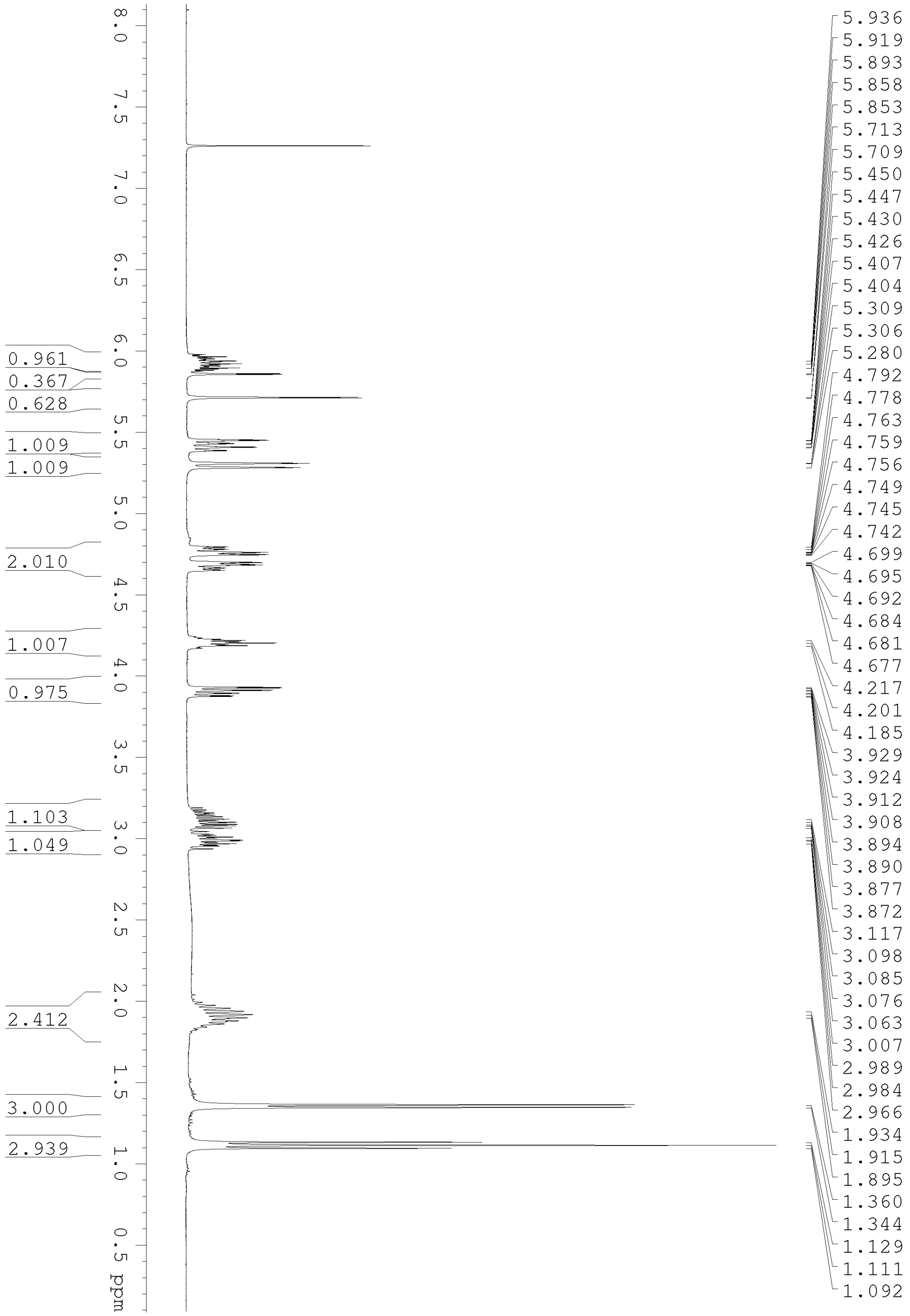

^{13}C spectrum of compound **5**

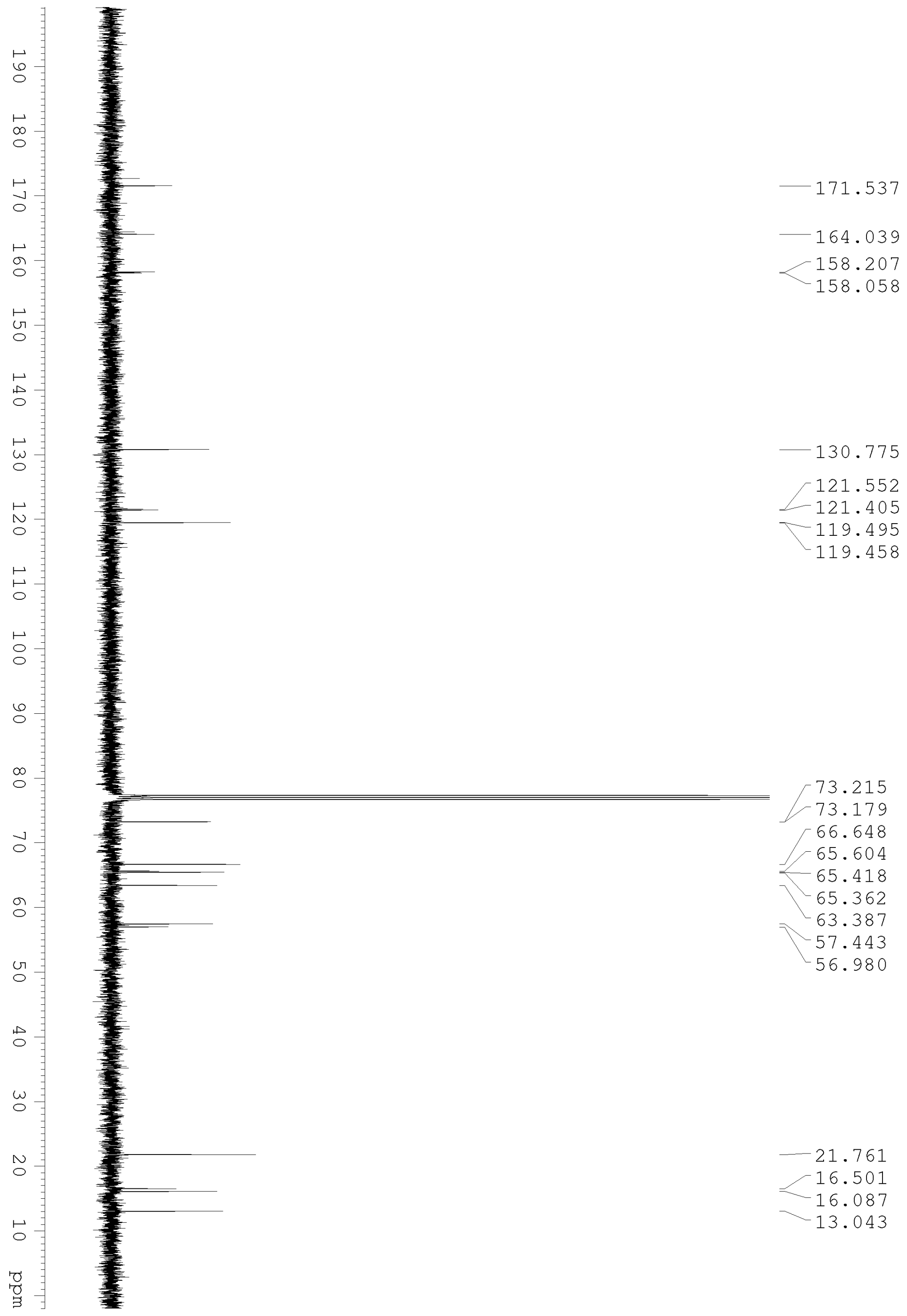

^1H spectrum of compound **6**

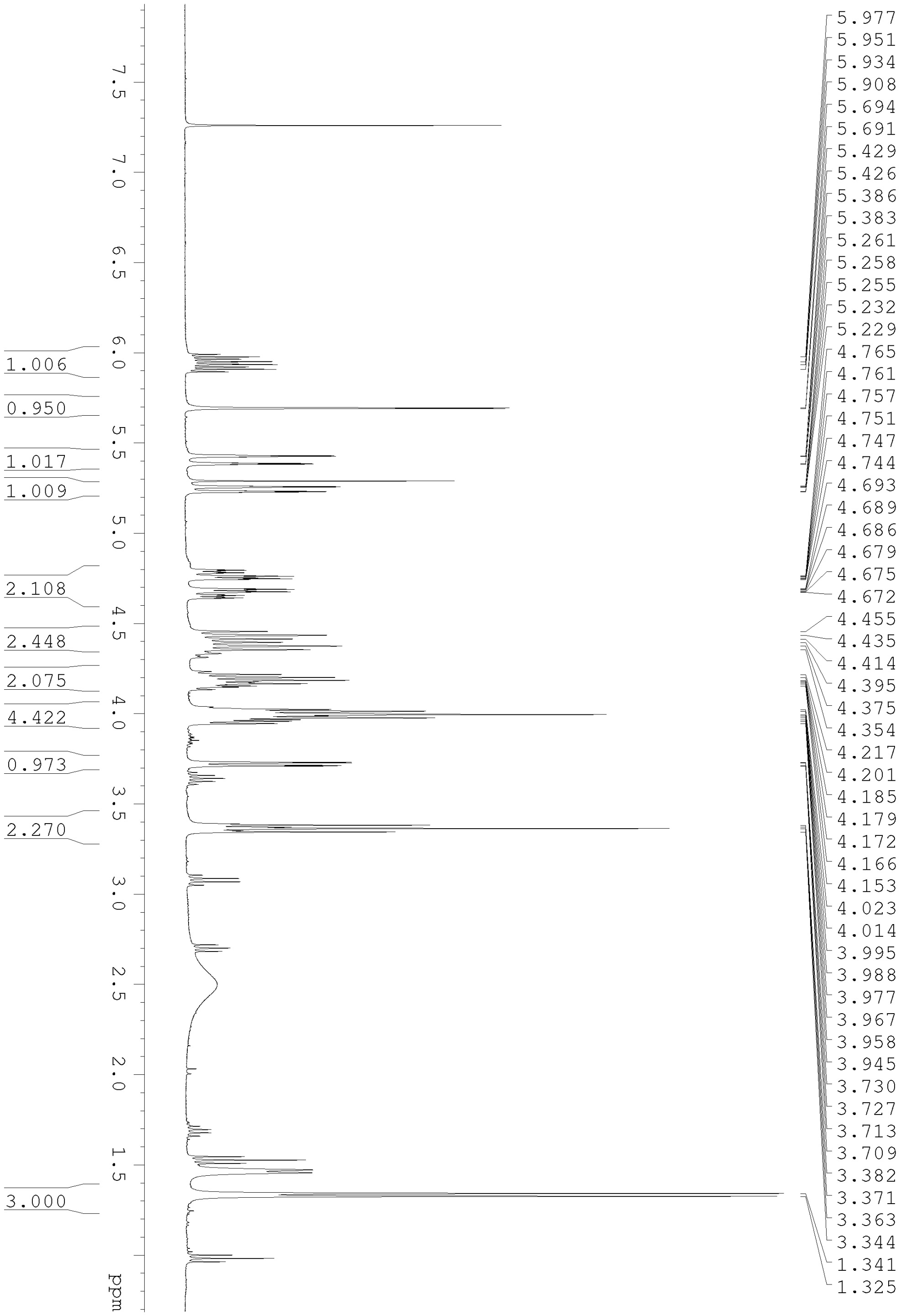

^{13}C spectrum of compound **6**

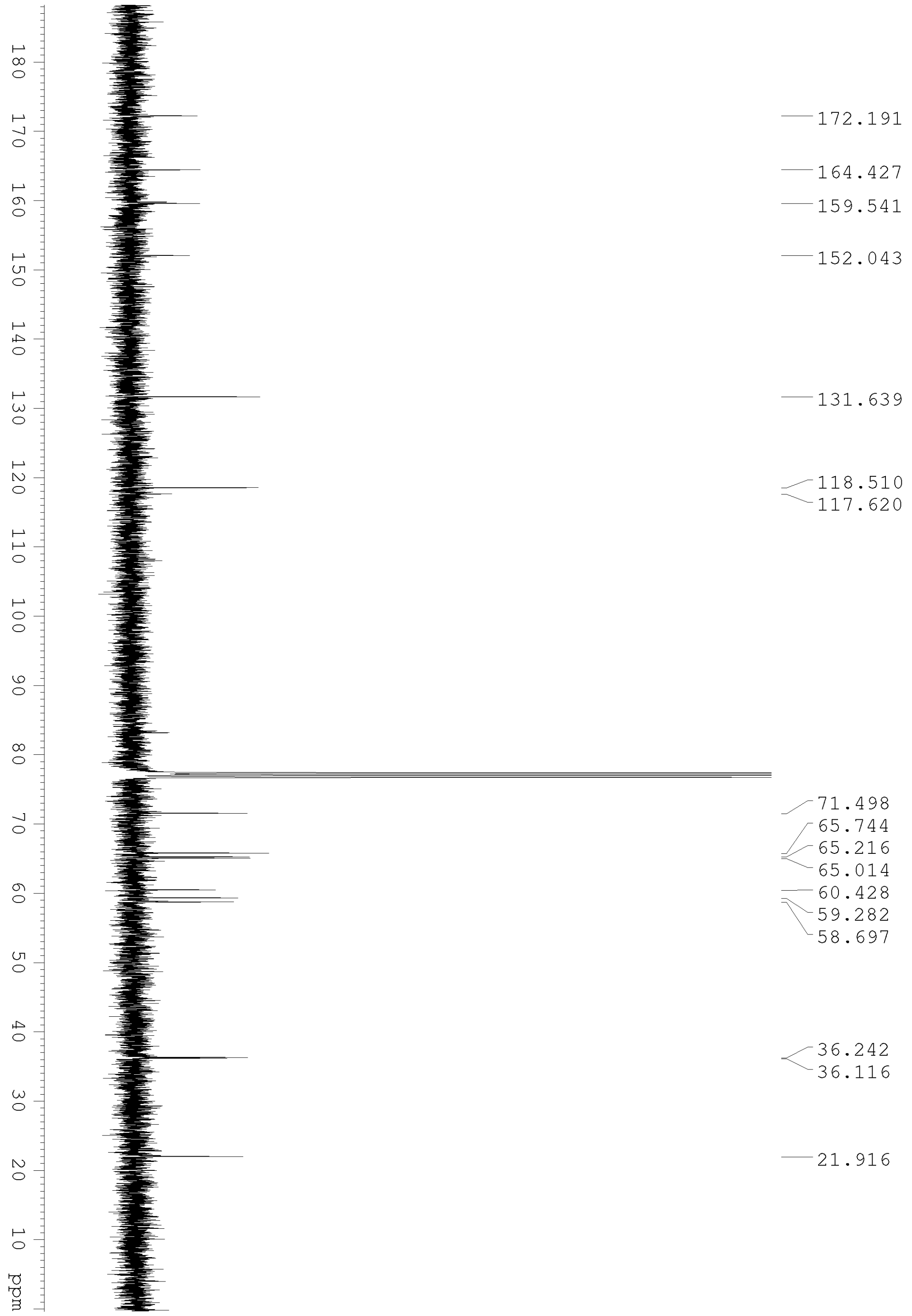

^{1}H spectrum of compound **T405**

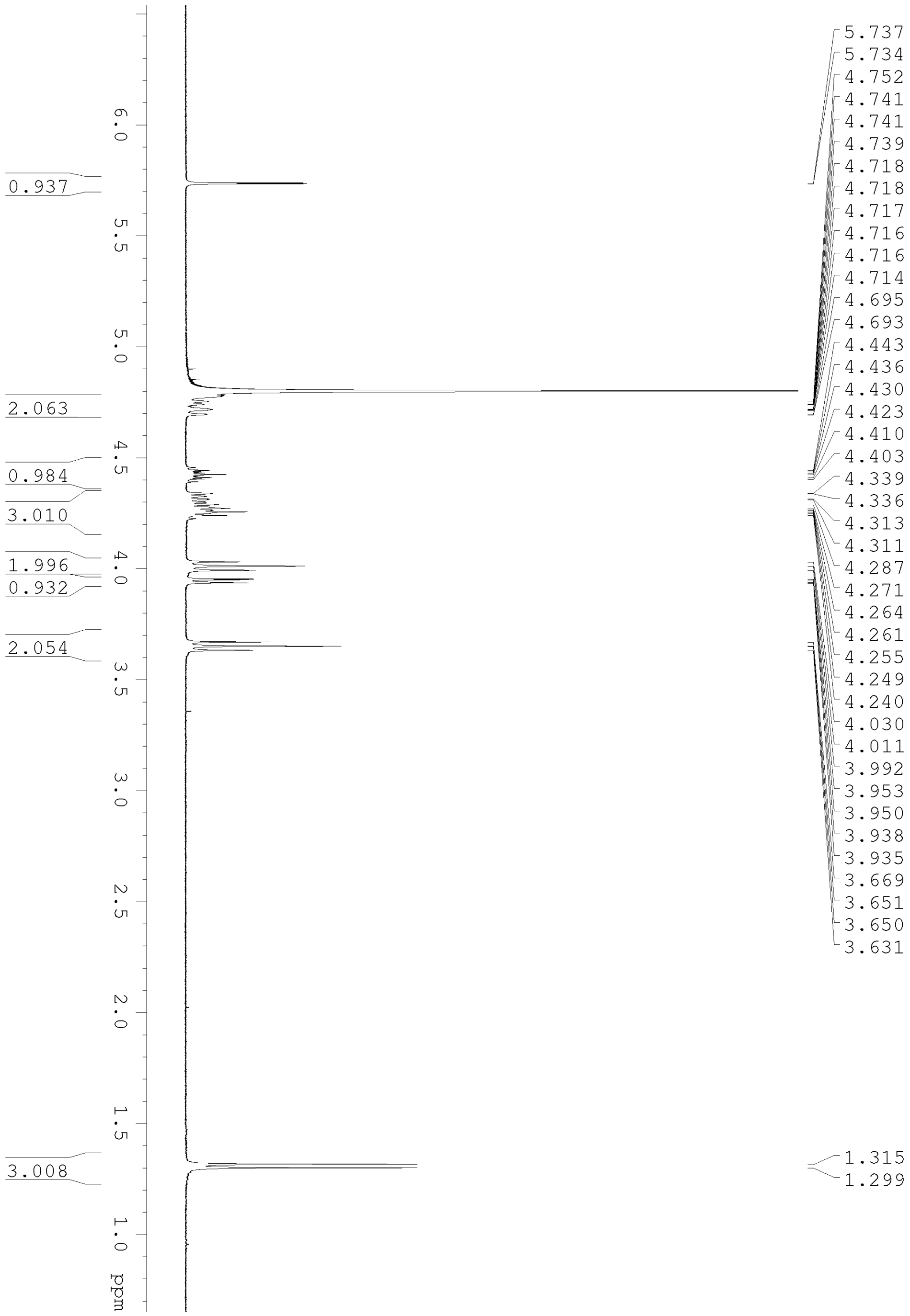

^{13}C spectrum of compound **T405**

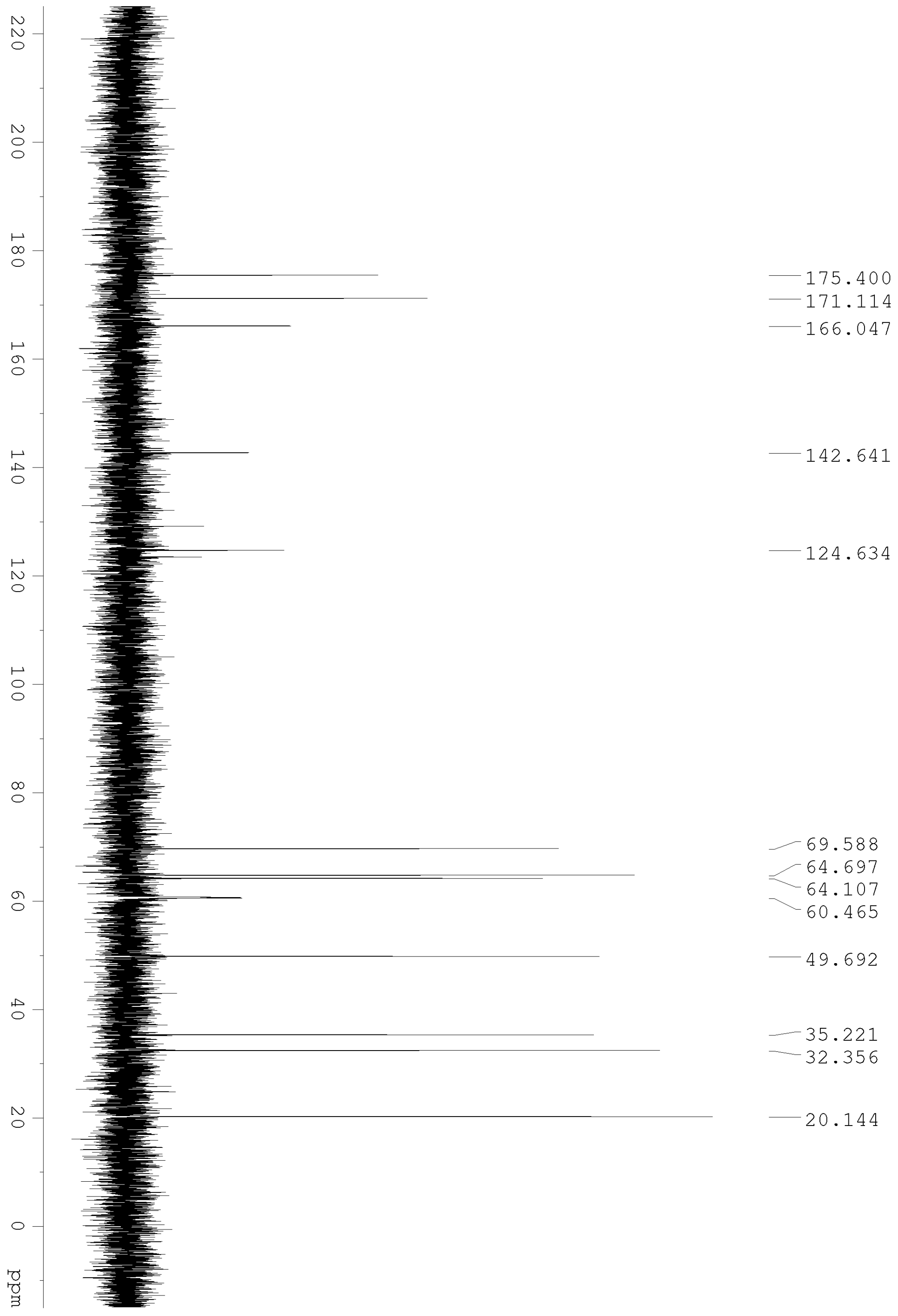

Supplement: Supplementary file 1 — Supplementary Information [file 42003_2020_1475_MOESM1_ESM.pdf]
